# 

**Published:** 1861-03

**Authors:** 


### CONTENTS.

### ORIGINAL COMMUNICATIONS.

PAGE.

ARTICLE I.—Hemorrhage from the Uterus continually during pregnancy. By P. M. Tidwell, M.D., of Fairburn, Ga., .......... 379

ARTICLE II.—Case of Strangulated Inguinal Hernia, occurring unexpectedly in an old ruptured subject. By Thomas S. Duffy, M.D., of Rutherfordton, N. C., ....................... 383

### SELECTIONS.

Diseases of the Urinary Organs. On the Tests for Albumen in the Urine. By Dr. Lionel Beale, F. R. S., Physician to King's College Hospital, &c.................... 386
A sketch of an Epidemic of Dengue or Breakbone Fever, as it prevailed in Wilmington, N. C., in the Autumn of the year 1860. By James H. Dickson, M.D.................... 394
Strychnia in "Hay Fever" and Influenza. By James Barrach, M.D., of Philadelphia,.................... 399
On what is commonly called "Rigidity of the Os Uteri." By Charles D. Arnott, M.D., Edin., &c.................... 402
Case of Delirium Tremens treated with large doses of the Tincture of Digitalis, New Orleans, Charity Hospital, service of Prof. Flint,. 405
Case of Areolar Cancer of the Peritoneum, with Hœmatocle of the Right, and Encephaloid Disease of the Left Ovary. By J. Forsyth Meigs, M.D., Physician to the Pennsylvania Hospital,....... 407
Therapeutical and Pharmaceutical Notes on Cimicifuga. By Edward Parrish,.................... 414
A New Test for Diabetis. By E. C. Bidwell, M.D.................... 417
On Hops and Lupulin. By Charles A. Tufts, of Dover, N. H......... 418
A few Thoughts on the Importance of Prophylactic Treatment in the Decay of Human Teeth. By Abr. Robertson, D.D.S., M.D., of Wheeling, Va.................... 420
Croup—Tracheotomy—Recovery.................... 425
Operation for the Removal of an Ovarian Tumor, weighing 17 lbs. Death of Patient by subsequent Internal Hemorrhage. By E. S.
Medical Society of Georgia.................... 436
Hydrophobia after Fourteen Month's Incubation,.................... 436
Whooping Cough,.................... 437
Chilblain,.................... 437
Book Notices and Items.................... 438 439
Precocity.................... 439
List of Payments,.................... 440

